# Supplementary material for: How can we make self-sampling packs for sexually transmitted infections and bloodborne viruses more inclusive? A qualitative study with people with mild learning disabilities and low health literacy
Source: Sex Transm Infect. 2021 Apr 27;97(4):276–81. doi: 10.1136/sextrans-2020-054869 (PMC8165145; doi:10.1136/sextrans-2020-054869)
Supplement: Supplementary data [file sextrans-2020-054869supp002.pdf]

LUSTRUM
Limiting Undetected Sexually Transmitted Infections to RedUce Morbidity

Version 1, 1 September 2017

Appendix 11: "Testing a sexual health treatment pack"

Study 5

Group discussion - Consent Form

Researchers: Dr Maria Pothoulaki, Mr Alan Middleton, Professor Paul Flowers (Glasgow Caledonian University)

Please tick the boxes below if you agree

I have read and understood the information sheet about the study (dated 1 September 2017, version 1). ☐

I have had the opportunity to ask questions ☐

My age is between 18 and 65 years of age ☐

I understand that if I want I can leave the group discussion at any point ☐

GCU Glasgow Caledonian University

Funded by NHS National Institute for Health Research

LUSTRUM
Limiting Undetected Sexually Transmitted Infections to RedUce Morbidity

Version 1, 1 September 2017

I agree to the group discussion being audio-recorded ☐

I agree for researchers to use my direct words (quotations) in publications, reports and/or presentations, without revealing my name. ☐

I understand that my personal details will not be shared outside the research team ☐

I understand that the only reason for sharing my details is if I am in danger and need help to keep me safe ☐

I understand the information about the study and I can decide to take part ☐

I agree to take part in the study ☐

_______________	_______________	_______________
Please write your name	Date	Please sign above here
_______________	_______________	_______________
Researcher's name	Date	Researcher's signature

GCU Glasgow Caledonian University

Funded by NHS National Institute for Health Research
