# Supplementary material for: How can we make self-sampling packs for sexually transmitted infections and bloodborne viruses more inclusive? A qualitative study with people with mild learning disabilities and low health literacy
Source: Sex Transm Infect. 2021 Apr 27;97(4):276–81. doi: 10.1136/sextrans-2020-054869 (PMC8165145; doi:10.1136/sextrans-2020-054869)
Supplement: Supplementary data [file sextrans-2020-054869supp003.pdf]

Version 1, 1 September 2017

Appendix 3: "Maximising intervention reach to wider populations: Testing the APT intervention pack"

INTERVIEW SCHEDULE

Study 5

I. Introduction

- *Introduction of the facilitator/researcher and participant*
- *Explain information about the study, the purpose of the interview and the type of questions that will be asked*
- *Explain that the participant is free to leave at any point*
- *Explain the use of the audio-recorder and how data will be handled*
- *Explain what type of information/data will be shared and with whom (anonymity and confidentiality)*
- *Check understanding of information, ask if participant has any questions and take informed written consent*
- *Ask Demographic questions*

  (Audio-recorder on)

II. Presentation of the Accelerated Partner Therapy (APT) intervention pack

- *Presentation of visual aid (APT Pack) and a brief background summary of how it will be used*

III. Assessing the strengths and weaknesses of the APT pack

- *Ask participant to open the pack, have a look at the contents of the pack and voice their thoughts*
- *Ask participants to read carefully the instructions in the pack*
- How do you think about the pack?
- What are the contents in this pack?
- How are the contents used?

Funded by NHS National Institute for Health Research

Version 1, 1 September 2017

Example of questions to be asked for each of the items in the APT pack

- Why is the Genital chlamydia information placed in the pack?
- What is good about it?
- What is bad about it?
- How could it be better?

(Ask the above questions for each of the APT pack items: condoms, instruction leaflet on how to use the pack, medication, urine collection container, blood collection pack, envelope, security seal, red label, laboratory form.)

- If you were to use the pack, what would you find most difficult?
- If you were to use the pack, what would you find most easy?
- What/who would help in using the pack?
- What would you recommend to the people who make the pack?

IV. Conclusion

Check if the participant has any questions or comments.

Thank you for participating in our study!

Funded by NHS National Institute for Health Research
